# An aspect-level sentiment analysis dataset for therapies on Twitter

**DOI:** 10.1016/j.dib.2023.109618

**Published:** 2023-09-23

**Authors:** Yuting Guo, Sudeshna Das, Sahithi Lakamana, Abeed Sarker

**Affiliations:** Emory University, Atlanta, Georgia 30322, USA

**Keywords:** Text classification, Sentiment analysis, Therapy, Natural language processing, Machine learning, Biomedical informatics

## Abstract

The dataset described is an aspect-level sentiment analysis dataset for therapies, including medication, behavioral and other therapies, created by leveraging user-generated text from Twitter. The dataset was constructed by collecting Twitter posts using keywords associated with the therapies (often referred to as treatments). Subsequently, subsets of the collected posts were manually reviewed, and annotation guidelines were developed to categorize the posts as positive, negative, or neutral.

The dataset contains a total of 5364 posts mentioning 32 therapies. These posts are further categorized manually into 998 (18.6%) positive, 619 (11.5%) negatives, and 3747 (69.9%) neutral sentiments. The inter-annotation agreement for the dataset was evaluated using Cohen's Kappa score, achieving an 0.82 score.

The potential use of this dataset lies in the development of automatic systems that can detect users' sentiments toward therapies based on their posts. While there are other sentiment analysis datasets available, this is the first that encodes sentiments associated with specific therapies. Researchers and developers can utilize this dataset to train sentiment analysis models, natural language processing algorithms, or machine learning systems to accurately identify and analyze the sentiments expressed by consumers on social media platforms like Twitter.

Specifications Table

**Table:** 

Subject	Artificial Intelligence
Specific subject area	Sentiment analysis is a subtopic within natural language processing that focuses on characterizing the sentiment expressed in a body of text.
Data format	Raw, Analyzed, Filtered
Type of data	Text
Data collection	The dataset was created by collecting Twitter posts using keywords associated with 32 therapies via Twitter's academic streaming application programming interface (API). Subsequently, subsets of the collected posts were randomly sampled and manually reviewed by four annotators based on annotation guidelines that were developed over multiple iterations. Finally, the annotated dataset was split into training, validation, and test sets to enable comparative analyses of machine learning approaches.
Data source location	Institution: Emory University City/Town/Region: Atlanta, Georgia Country: United States of America Latitude and longitude (and GPS coordinates, if possible) for collected samples/data: N/A
Data accessibility	Repository name: Zenodo Data identification number: 10.5281/zenodo.8186910 Direct URL to data: https://zenodo.org/record/8186910 Instructions for accessing these data: The data can be accessed via the Twitter Academic API [1]. Detailed instructions are provided in the readme.txt file accompanying the data in the abovementioned links.
Related research article	N/A

## 1 Value of the Data

• Sentiment analysis is a natural language processing (NLP) technique used to determine the emotional tone or sentiment expressed in a piece of text. It involves using computational methods to automatically identify and classify the sentiment as positive, negative, neutral. There is a paucity of data with annotations describing consumer sentiments about therapies, and this dataset aims to address that deficiency by providing such annotations.
• This dataset will be valuable for natural language processing and machine learning researchers interested in building models for automated, aspect-oriented sentiment analysis of consumer-generated texts associated with therapies. It will also be valuable for medical domain experts interested in the application of automated methods for understanding sentiments associated with specific therapies.
• Researchers and developers can utilize this dataset to train sentiment analysis models, natural language processing algorithms, or machine learning systems to accurately identify and analyze the sentiments expressed by consumers on social media platforms like Twitter. Medical domain experts can leverage trained models to study sentiments associated with targeted therapies.

## 2 Data Description

This dataset is an aspect-level sentiment analysis dataset for therapies, created by leveraging user-generated text from Twitter. The dataset contains a total of 5364 posts related to 32 therapies, including medications, behavioral, and physical therapies. These posts are further categorized into 998 (18.6%) positive, 619 (11.5%) negative, and 3747 (69.9%) neutral sentiments. The inter-annotator agreement for the dataset was evaluated using Cohen's Kappa score [2], achieving an 0.82 score, which represents substantial agreement [3]. The data was split into training, validation, and test sets. The training set contained 3762 annotated posts, including 753 posts for validation: 718 (19%) with positive sentiment, 413 (11%) with negative sentiment, and 2631 (70%) with neutral sentiment. The test set contained 1602 annotated posts: 280 (17%) with positive sentiment, 206 (13%) with negative sentiment, and 1116 (70%) with neutral sentiment. The data repository contains three CSV files named “train_id_only.csv,” “dev_id_only.csv,” and “test_id_only.csv,” which serve as the training, validation, and test sets. These datasets include three essential fields: “tweet_id,” “therapy,” and “label.” The “tweet_id” field contains unique identifiers that enable access to the post text through the Twitter application programming interface (API). The “therapy” field specifies the therapy-related keyword mentioned in each post, which serves as the target for sentiment analysis. The “label” field contains human-annotated sentiment labels associated with the mentioned therapy. To uphold Twitter's privacy policy, we have chosen to provide only the post IDs and not the actual text content. Researchers interested in the data are required to utilize the post IDs with the Twitter API to retrieve the post text. Table 1 provides examples illustrating the structure of these datasets along with the associated post text.

**Table 1:** The examples of the posts which showed neutral, positive, and negative sentiments towards meditation, massage, and hydrocodone, respectively.

tweet_id	therapy	text (sample)	label
1557073922629242880	meditation	A New Chronic Pain Treatment: Meditation Can Reduce Your Pain by 32% // In my personal experience, pain is pain but the article is interesting and worth the read.<url> <usr> #meditation #mindfulness #chronicpain	neutral
1559960890291658755	massage	Gotta admit, I'm as addicted to a monthly deep tissue massage as I am to the Humira to manage this AS! So relaxing! #spoonie #ChronicPain	positive
1555908688979820545	hydrocodone	Hey y'all. I need some help. My MIL has been dealing with chronic pain for years now. She's been taking prescription hydrocodone but it hasn't helped. She's been supplementing that with wine.	negative

## 3 Experimental Design, Materials and Methods

To collect relevant data, we employed a targeted approach by retrieving posts from Twitter using a carefully curated list of keywords associated with therapies, including medication, behavioral and physical therapies. The collected posts underwent a meticulous manual review process to ensure data quality. Furthermore, annotation guidelines were developed to classify the posts into three sentiment categories: positive, negative, and neutral. Using the Twitter Academic API, we collected posts mentioning at least 32 therapies. Since medication names are often misspelled on social media, we used an automatic misspelling generator to obtain common misspellings for the therapy names. We collected the posts between May 2021 and August 2023 accruing 171,961 related posts and randomly sampled a subset for annotation.

Subsequently, subsets of the collected posts were manually reviewed, and annotation guidelines were developed to categorize the posts as positive, negative, or neutral. As the first step, 100 posts were labelled by two annotators to determine the sentiment related to the therapies mentioned in the posts. The initial inter annotator agreement (IAA) between the two annotators was 0.20 (Cohen's kappa). To optimize the agreement between annotators, we developed an annotation guideline to determine whether posts should be labelled as positive, negative, or neutral. After the guideline was implemented, we extracted additional 200 posts. The same two annotators annotated these posts, and the IAA improved to 0.32. After further optimizing the guidelines, we extracted additional 200 posts and a third annotator also annotated the posts. The average IAA between three annotators improved to 0.42. We analyzed the disagreements, resolved them via discussion, and updated the annotation guideline accordingly. Finally, an additional 200 posts were annotated by 4 annotators based on the final guideline, and the final IAA was computed to be 0.82.

Upon achieving acceptable agreement, a total of 5364 posts were annotated based on the same guideline. The finalized annotation guidelines require that label a post as positive or negative only if the post (i) mentions a therapy word in the context of therapy, (ii) shows an explicit link between sentiment and therapy, and (iii) indicates that someone (user or others) has tried the therapy. The full guidelines are provided as supplementary material. Finally, the annotated dataset was split into training, validation, and test sets to enable comparative analyses of machine learning approaches. The detailed statistics of the dataset are shown in Table 2.

**Table 2:** The data sizes and class distributions of the training, validation, and test sets.

DATA	SIZE	POSITIVE	NEGATIVE	NEUTRAL
TRAINING SET	3009	570 (18.9%)	319 (10.6%)	2120 (70.5%)
VALIDATION SET	753	148 (19.7%)	94 (12.5%)	511 (67.8%)
TEST SET	1602	280 (17.5%)	206 (12.8%)	1116 (69.7%)
TOTAL	5364	998 (18.6%)	619 (11.5%)	3747 (69.9%)

## 3 Limitations

In our dataset, each post was labeled with only one therapy. However, it is not uncommon for posts to mention multiple therapies simultaneously. For instance, users may compare different therapies within a single post. Annotating the sentiment for each therapy keyword in a post can be a resource-intensive task. Considering the constraints of time and budget, we opted to annotate only one therapy per post.

## Ethics Statement

**Terms of Service (ToS):** Regarding the web resource used for data collection, we have carefully considered and adhered to the respective Terms of Service, Privacy Laws, and any necessary consents. We ensure compliance with these legal and ethical considerations when scraping and distributing the data.

**Copyright:** Twitter users retain their rights to the post content and grant Twitter a license to distribute the content. We respect copyright laws and terms of use in handling and sharing the data.

**Privacy:** We recognize the importance of privacy concerns and have taken measures accordingly. Depending on the context, we have followed recommended guidelines for preserving user privacy when sharing non-anonymized data.

Scraping Policies: We have considered and followed any special scraping policies that may be in place on the web resource, such as those imposed by platforms like Twitter. Our data were collected via Twitter Academic API, and the collection methods adhere to these policies and are conducted in a manner consistent with ethical research practices.

## CRediT authorship contribution statement

**Yuting Guo:** Methodology, Writing – original draft. **Sudeshna Das:** Data curation, Writing – review & editing. **Sahithi Lakamana:** Software. **Abeed Sarker:** Conceptualization, Supervision, Writing – review & editing.

## Data Availability

An Aspect-Level Sentiment Analysis Dataset for Therapies on Twitter (Original data) (Zenodo).

## Appendix Supplementary materials

Image, application 1
