# Supplementary material for: An aspect-level sentiment analysis dataset for therapies on Twitter
Source: Data Brief. 2023 Sep 23;50:109618. doi: 10.1016/j.dib.2023.109618 (PMC10558704; doi:10.1016/j.dib.2023.109618)
Supplement: Supplementary file 1 [file mmc1.docx]

Sentiment annotation guideline

Each tweet mentions a therapy, which can be a medication or another form of therapy. The objective is to annotate the sentiment of each tweet associated with the given therapy. The therapy is mentioned under the **Therapy** column. This column is useful when multiple therapies are mentioned in the tweet.

Label each tweet as follows:

- Positive sentiment associated with a therapy: 1
- Neutral sentiment: 0
- Negative sentiment associated with therapy: -1

# Annotation rules

It is expected that most tweets express a neutral sentiment. Label a tweet as positive or negative only if it meets all of hese requirements:

1. Mentions a therapy word in the context of therapy.
2. Shows an explicit link between sentiment and therapy.
3. Indicates that someone (user or others) has tried the therapy.

The following tables describe more detailed guidelines for neutral, negative, positive cases, and other cases.

## 1. Neutral cases

| **Rule** | **Example** |
| --- | --- |
| 1.1 If the context word is not mentioned as a therapy, the label should be neutral. | Therapy: *aquatic*  Post: *the closest I came to a pleasant experience with the ket was day 3, which felt very... um, I want to say "****aquatic****"? like I was in an egg that was floating. I think the music contributed to that a lot, but the same music yesterday made me sob. so. :') idk.*  Label: *0* |
| 1.2 If there is no explicit evidence that the user has actually tried the therapy, the label should be neutral. | Therapy: *massage*  Post: *ordered a* ***massage*** *gun and an acupressure cushion for my back and i can’t remember the last time i was so excited lmao*  Label: *0* |
| 1.3 If there is implicit positive/negative sentiment associated with the therapy, but it is not clear enough, the label should be neutral. | Therapy: *tramadol*  Post: *Health update: I was apprehensive about seeing my GP again today, but he was way more pliant than last time. With little fuss, I got* ***tramadol*** *&amp; more prednisolone. Without the latter, I have symptoms not much less severe than my bout of Covid, *all* the time. I might survive now.*  Label: *0* |
| 1.4 If the sentiment is clear enough, but there is no explicit or implicit link between the sentiment and therapy, the label should be neutral. | Therapy: *hydrocodone*  Post: *Pain management today: only take your* ***hydrocodone*** *PRN.. excuse me. I do take it only when I need it. Smdh. Then tells me not to take it bc "we don't know where my pain is coming from".. again from EDS!!! Told to stop taking meds and go to therapy 🙄 they just don't get it*  Label: *0* |

## 2. Negative cases

| **Rule** | **Example** |
| --- | --- |
| 2.1 If the therapy is not effective, the label should be negative | Therapy: *tramadol*  Post: *Oh my god, I am in so much pain tonight. My face feels like I've been hit with a baseball bat. I've taken* ***tramadol*** *and it isn't touching the pain. Cover up in the sun. Get any suspicious skin spots checked. Don't wait before seeking medical attention.*  Label: *-1* |
| 2.2 If it is mentioned that the person does not want the therapy, the label should be negative. | Therapy: *Botox*  Post: *I was supposed to have my next round of Botox injections this morning for intractable migraines. This would be my 5th round. But I cancelled them because I was scared.*  Label: *-1* |
| 2.3 The sentiments toward the side effects and therapeutic effect of a therapy should both be considered. When there is negative sentiment referring to side effects of the therapy, but it is not clear if the person is still willing to continue the therapy for its therapeutic effect, the label should be negative. | Therapy: *emgality*  Post: *i injected my stomach with* ***emgality*** *last night and it is still fucking itching 😬*  Label: *-1* |

## 3. Positive cases

| **Rule** | **Example** |
| --- | --- |
| 3.1 Any improvement will be considered positive sentiment, unless the person decided that the improvement is too small to continue the therapy | Therapy: *Botox*  Post: ***Botox*** *for migraine has reduced mine by about %60 but, Imitrex works better than Nurtec for me acute migraine but does help for prevention. ( for JVNs spaces discussion)*  Label: *1* |
| 3.2 The sentiment toward a therapy’s side effect and therapeutic effect should both be considered. When there’s negative sentiment about the side effects of the therapy but the person is still willing to continue the therapy for its positive therapeutic effect, the label should be positive. | Therapy: *topiramate*  Post: *topamax has impaired my vision and I cannot walk in a straight line and I got the shakes and it makes me stupid BUT that shit WORKS! LIKE THAT SHIT WORKKKKKKKSSSS. MY MIGRAINES ARENT EVEN A PROBLEM!!!!!!!! THE SIDE EFFECTS MAY SUCKS FOR A BIT BUT ITS BETTER THAN THE MIGRAINES!!!*  Label: *1* |

## 4. Other cases

| **Rule** | **Example** |
| --- | --- |
| 4.1 If it is about a 3rd person like a family member or friend, we still look at the sentiment associated with the therapy in context. | Therapy: *massage*  Post: *Bakugou's palms often hurt after he overused his quirk all day. Kirishima was here to* ***massage*** *his palms with healing ointment.*  Label: *0* |
| 4.2 When judging the sentiment, emojis and hashtags should be considered. | Therapy: *meditation*  Post: *Morning filled with* ***meditation****, breath work, and singing along to good music 🥰😍🔥*  *Now it’s time to eat good food and get to work 👩‍💻✨.*  Label: *1* |
